# Associations between neighbourhood fast-food environments and hypertension in Canadian adults.

**DOI:** 10.23889/ijpds.v7i3.2082

**Published:** 2022-08-25

**Authors:** Jacqui Oakley, Robin Flaig, Emma Turner, Kirsteen Campbell, Katharine Evans, Stela McLachlan, Richard Thomas

**Affiliations:** 1 Department of Geography, McGill University, Montréal, Quebec; 2 Health Analysis Division, Statistics Canada, Ottawa, Ontario; 3 Department of Epidemiology; Department of Medicine; Centre for Outcomes Research and Evaluation, McGill, Montréal; 4 School of Planning, University of Waterloo, Waterloo, Ontario; 5 Department of Geography; Institute for Health and Social Policy, Faculty of Medicine, McGill, Montreal, Quebec; 6 Department of Geography and Planning, University of Toronto, Toronto, Ontario; 7 Queen’s University, Kingston, Ontario

## Objectives

Hypertension is a leading cause of cardiovascular disease and premature death. Neighbourhoods characterized by a high proportion of fast-food outlets may contribute to hypertension in residents; however, limited research has explored these associations. The objectives of this study were to assess associations between neighbourhood fast-food environments, measured and self-reported hypertension.

## Approach

We used data from 10,700 adults who participated in six cycles of the Canadian Health Measures Survey (CHMS). Measured hypertension was defined as having an average systolic blood pressure (BP) of ≥140, a diastolic BP ≥90 mm Hg or being on BP lowering medication. Participants were also asked if they had been diagnosed with high BP or if they take BP lowering medication (i.e., self-reported hypertension). We characterized the fast-food environment of each participant’s neighbourhood using the Canadian Food Environment Dataset (Can-FED). We considered the proportion of fast-food outlets relative to fast-food outlets and full-service restaurants as a continuous variable.

## Results

The mean proportion of fast-food outlets was 23.3% (SD 26.8%). A one standard deviation (SD) increase in the proportion to fast-food outlets was associated with higher odds of measured hypertension in the full sample (OR=1.17, 95% CI 1.05 to 1.31) and in sex-specific models (women: OR=1.14, 95% CI 1.01 to 1.29; and men: OR=1.21, 95% CI 1.03 to 1.43). A one standard deviation (SD) increase in the proportion to fast-food outlets was associated with higher odds of self-reported hypertension in the full sample (OR=1.13, 95% CI 1.02 to 1.24); however, associations were inconclusive in sex-specific models (women: OR=1.11, 95% CI 0.99 to 1.26; and men: OR=1.14, 95% CI 0.99 to 1.33).

## Conclusion

By linking neighbourhood food environment measures that were created from an administrative data source (the Statistics Canada Business Register) to individual-level data from the CHMS, we were able to demonstrate that reducing the proportion of fast-food outlets in neighbourhoods may reduce rates of hypertension and support individually targeted interventions.

